# The emergence of proton nuclear magnetic resonance metabolomics in the cardiovascular arena as viewed from a clinical perspective

**DOI:** 10.1016/j.atherosclerosis.2014.09.024

**Published:** 2014-11

**Authors:** Naomi J. Rankin, David Preiss, Paul Welsh, Karl E.V. Burgess, Scott M. Nelson, Debbie A. Lawlor, Naveed Sattar

**Affiliations:** a BHF Glasgow Cardiovascular Research Centre, University of Glasgow, Glasgow, G12 8TA, UK; b Glasgow Polyomics, Joseph Black Building, University of Glasgow, Glasgow, G12 8QQ, UK; c School of Medicine, University of Glasgow, Glasgow, G12 8TA, UK; d MRC Integrative Epidemiology Unit at the University of Bristol, Bristol, BS8 2BN, UK; e School of Social and Community Medicine, University of Bristol, Bristol, BS8 2PS, UK

**Keywords:** Nuclear magnetic resonance (^1^H NMR), Metabolomics, Cardiovascular disease (CVD), Lipoprotein, Mass spectrometry (MS), Biomarkers, Advanced lipoprotein testing (ALP)

## Abstract

The ability to phenotype metabolic profiles in serum has increased substantially in recent years with the advent of metabolomics. Metabolomics is the study of the metabolome, defined as those molecules with an atomic mass less than 1.5 kDa. There are two main metabolomics methods: mass spectrometry (MS) and proton nuclear magnetic resonance (^1^H NMR) spectroscopy, each with its respective benefits and limitations. MS has greater sensitivity and so can detect many more metabolites. However, its cost (especially when heavy labelled internal standards are required for absolute quantitation) and quality control is sub-optimal for large cohorts. ^1^H NMR is less sensitive but sample preparation is generally faster and analysis times shorter, resulting in markedly lower analysis costs. ^1^H NMR is robust, reproducible and can provide absolute quantitation of many metabolites. Of particular relevance to cardio-metabolic disease is the ability of ^1^H NMR to provide detailed quantitative data on amino acids, fatty acids and other metabolites as well as lipoprotein subparticle concentrations and size. Early epidemiological studies suggest promise, however, this is an emerging field and more data is required before we can determine the clinical utility of these measures to improve disease prediction and treatment.

This review describes the theoretical basis of ^1^H NMR; compares MS and ^1^H NMR and provides a tabular overview of recent ^1^H NMR-based research findings in the atherosclerosis field, describing the design and scope of studies conducted to date. ^1^H NMR metabolomics-CVD related research is emerging, however further large, robustly conducted prospective, genetic and intervention studies are needed to advance research on CVD risk prediction and to identify causal pathways amenable to intervention.

## Highlights

• ^1^H NMR metabolomics is being increasingly applied to large cohort studies.
• Studies have identified potentially novel lipoprotein and metabolite predictors for CVD.
• Potential exists for the use of metabolomics in cardiovascular clinical practice.
• Current findings are too preliminary to translate into clinical recommendations.
• Further large scale studies are now needed to advance the field in a robust manner.

## 1 Introduction

The metabolome is the entire small molecule (metabolite) complement of a system. Metabolites are generally defined as having an atomic mass of less than 1.5 kDa [1], [2]. In humans, these metabolites can be exogenous (e.g. dietary or drug related), endogenous (substrates, intermediates and final products of chemical reactions), and derived from the effect of the microbiome. Metabolites include carbohydrates, peptides, lipids, nucleotides, amino acids, organic acids and many other classes of small molecule [3], [4].

Metabolomics is the use of analytical chemistry methods combined with chemometrics for the study of the metabolome. Chemometrics, in turn, is the application of statistical and computational methods to extract data from experimentally derived spectra. The two most commonly used methods of probing the metabolome are: mass spectrometry (MS) and proton nuclear magnetic resonance (^1^H NMR).

There are two ways of quantifying the metabolites in a metabolomics experiment, termed absolute and relative quantitation [5]. For relative quantitation the (normalised) instrument response to the metabolite(s) is used to obtain a measure of that metabolite which can be compared within that cohort or batch [5]. However, as these are not in SI units, it is difficult to compare groups to other studies or, within the cohort, fully understand the clinical importance of results. The second way of quantifying metabolites, absolute quantitation, is more stringent [5]: involving calibrators and numerous isotopically-labelled internal standards (IS) (depending on the method) [6].

There are two main methodological strategies for probing the metabolome: targeted and untargeted (global) methods [2], [7]. In targeted metabolomics a pre-defined subset of metabolites are chosen and a particular analytical method optimised for that subset is used [7]. In non-targeted metabolomics, the aim is to identify and quantify as many metabolites as possible [8], [9]. However, due to the diverse nature of metabolites in terms of their physio-chemical properties and dynamic range (ratio of highest versus lowest concentration: e.g. pM to mM) there is no single method that can detect all metabolites [3], [8]. Targeted methods report fewer metabolites and are more likely to be hypothesis driven.

Cardiovascular disease (CVD) remains the leading cause of death worldwide [10]. Hypertension, smoking, diabetes mellitus and dyslipidaemia are major risk factors for CVD [11] and are incorporated into risk scores. Such scores are important in assessing treatment needs for primary prevention and are widely used. However, such scores are not perfect and researchers are continually working to improve these scores [12]. It is hoped that methods that probe the metabolome and lipoprotein profile could potentially be used to identify novel biomarkers or pathways for atherosclerosis, improve clinical prediction of CVD, and investigate the metabolic consequences of specific therapies or interventions [8], [11], [13], [14].

This review will briefly outline the key methodological principles of ^1^H NMR. We focus on ^1^H NMR because of the recent advances with this method; its markedly lower cost in comparison to MS, resulting in an increasing number of clinically relevant studies using this technique, and the potential for clinical application, already being realised to some extent in the USA [9], [15], [16]. To illustrate the potential of ^1^H NMR technology we will review some early gains in the cardiometabolic arena from ^1^H-NMR-based studies. We also highlight the requirements that need to be met before ^1^H NMR is widely adopted in epidemiological research and, ultimately, applied to routine clinical care. Finally, we briefly describe the benefits and limitations of ^1^H NMR, making reference to MS as a comparator method. In so doing, we suggest the two methods provide complementary, rather than competing, methodologies.

## 2 Proton ^1^H NMR

### 2.1 The theoretical basis of proton ^1^H NMR

**Fig. 1:**
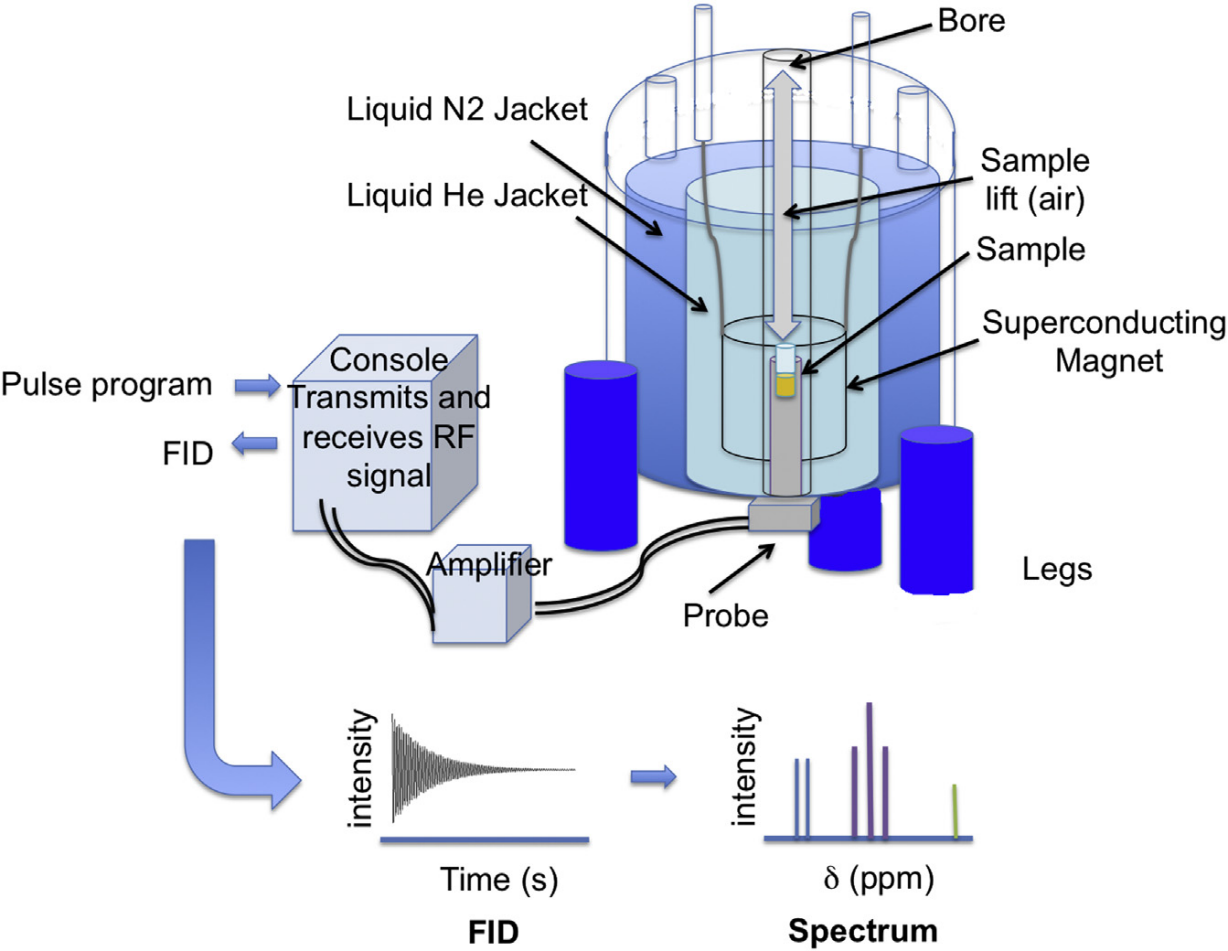
Simplified diagram of a nuclear magnetic resonance spectrometer. At the heart of the ^1^H NMR spectrometer is a superconducting magnet. This must be kept at 4 K, so needs to be emerged in liquid helium, which is prevented from evaporating by vacuum and nitrogen jackets. The probe, containing the RF coil sits in the bottom of the magnet within its bore. The sample is always contained within the ^1^H NMR tube; it is gently dropped into the probe on a cushion of air. Here the superconducting magnet causes the protons to spin and the RF coil sends RF pulses to excite them and collects the free-induction decay as they relax back to equilibrium. The pulse programs are created using the computer and sent to the console, which acts both as a radiofrequency transmitter and receiver. The signals are amplified on transmission and receipt. The FIDs are Fourier transformed (mathematically deconvoluted) to produce ^1^H NMR spectra of intensity versus chemical shift (*δ*) using the computer.

^1^H NMR spectroscopy is a technique that exploits the magnetic properties of protons in order to obtain information about the structure of a molecule, and hence its identity [17]. The sample is placed in a strong magnetic field and electromagnetic radiation, in the form of radiofrequency pulses, is used to excite the protons (Fig. 1). As the protons relax back to equilibrium the energy is recorded as an oscillating electromagnetic signal, called the free induction decay (FID). This is analogous to a number of bells ringing out after they have been simultaneously struck – each frequency of each bell will be overlaid and they will decay together. This complex waveform (intensity versus time) is normally Fourier Transformed (mathematically deconvoluted) in order to produce a spectrum of intensity versus frequency [18]. This is analogous to separating out the individual frequencies sounded by each bell, identifying what all those frequencies were and how loud each one was.

**Fig. 2:**
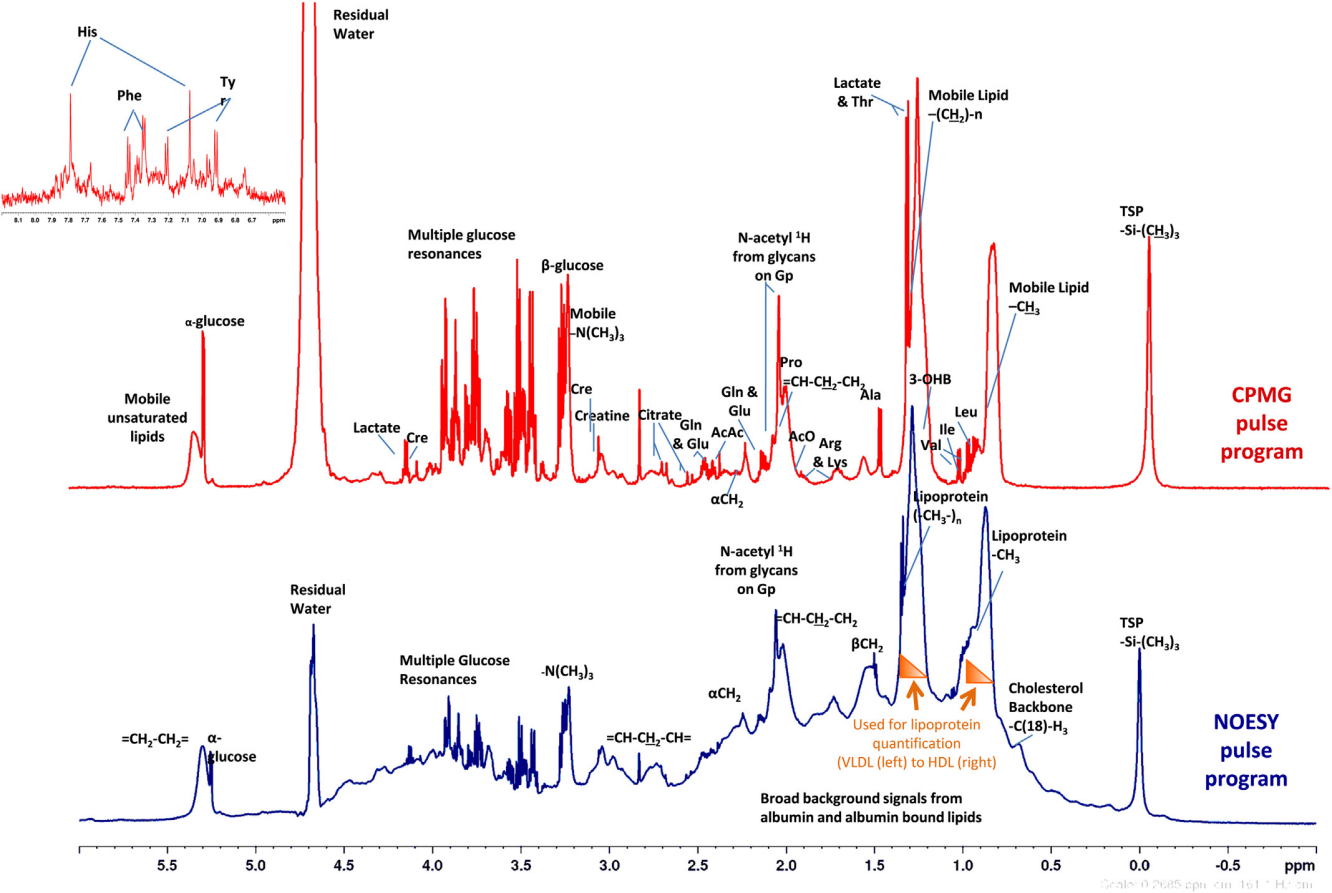
Typical ^1^H NMR spectra of serum analysed with two different pulse programs. Nuclear Overhauser Effect Spectroscopy (NOESY in blue) experiment used for Lipoprotein quantification and Carr–Purcell–Meiboom–Gill (CPMG in red) experiment used to quantify low molecular weight metabolites. Insert shows the aromatic region of the CPMG spectrum. Spectra were analysed and interpreted using the Finnish method (35, 42). The broad resonances arising from methy and methylene groups of lipoprotein lipids depend on the composition and size of the lipoprotein and can be deconvoluted to quantify lipoprotein subfractions. Key: TSP; 3-(trimethylsilyl)-2,2’,3,3’-tetradeuteropropionic acid; N-acetyl ^1^H from glycans on Gp; glycoprotein (mostly α-1-acid glycoprotein); Leu: leucine; Ile: isoleucine; Val: valine; Thr: threonine; 3-OHB: 3-hydroxybutyrate; Ala; alanine; Arg: arginine; Lys: lysine; AcO; acetate; Pro: proline; Gln: glutamine: Glu: glutamate; AcAc: acetoacetate; Cre: creatinine; His: histidine; Phe: phenylalanine; Tyr: tyrosine. (For interpretation of the references to colour in this figure legend, the reader is referred to the web version of this article.)

The data are represented as a spectrum of peaks with chemical shift (*δ*), in parts per million (ppm), along the *x*-axis and intensity along the *y*-axis. The chemical shift is the resonant frequency of the nucleus compared to the nucleus of an internal standard (IS), normally tetramethylsilane (TMS) or a related compound. The distance (in ppm) between the resonant frequency observed and the TMS signal depends on the chemical environment of the proton, i.e. the molecular structure. Different protons in different parts of the molecule have a different chemical shift and molecules give a specific pattern of peaks, in terms of both the chemical shift and the intensities of those peaks (Fig. 2). Quantitative ^1^H NMR (qNMR) is also achieved by comparison to the intensity of this reference peak (normally added to the sample at a known concentration), after taking into account the number of protons contributing to each peak.

^1^H NMR is a versatile method, with different pulse programs available for optimisation of large or small molecules by enhancing or attenuating different signals (Fig. 2). For example, the Carr–Purcell–Meiboom–Gill (CPMG) pulse program is used to identify small molecules in the presence of large proteins and lipoproteins [19]. In the CPMG sequence there is a longer delay between the excitatory pulse and the acquisition period. The large molecules (lipids and proteins) will have stopped spinning, whereas the small molecules are still spinning and producing an FID signal. This essentially renders the large molecules invisible on ^1^H NMR. An analogy would be striking two bells at the same time but only recording the sound once the bell with the shorter ring has stopped ringing.

### 2.2 Pre-analytical factors and sample preparation

Care must be taken in any biochemical assay, including metabolomics, to avoid bias or artefacts due to variation in sample collection, handling and storage. Standard operating procedures should be followed [20], [21]. The Metabolite Standards Initiative gives recommendations on the types of information that should be recorded, such as time of sampling, centrifugation and freeze–thaw cycles [5]. Samples for NMR metabolomics should be frozen immediately after processing (which should be completed within 2 h of sample collection) and long-term storage should be at ultra-low temperatures (−70 or −80 °C) [22]. Minimal differences between plasma (lithium heparin) and serum have been observed [22], [23]. Ethylene diamine tetraacetic acid (EDTA) and its complexes give contaminating peaks in ^1^H NMR plasma spectra but obscured signals in these regions can still be identified and quantified by signals in other spectral regions, with the exception of citrate, free choline and dimethylamine [23], [24]. The effect of different preparation and storage procedures vary by analyte and it is important to note that, in part because this field is relatively new, the effects are only recorded for a limited number of the metabolites that are increasingly quantified.

Serum or plasma samples are routinely mixed with buffer to minimise shifts due to pH. The buffer is usually inorganic and has deuterium oxide added for locking the magnetic field, imidazole as a pH indicator, azide as biocide and an IS for referencing the chemical shift and as a quantification standard [25].

### 2.3 Data analysis of spectra from ^1^H NMR in metabolomics

There are two broad ways of dealing with the spectra obtained from ^1^H NMR experiments.

1. **Metabolite Fingerprinting** (also known as the chemometric approach) uses the spectral pattern as a whole to determine the spectral features that are statistically different between sample classes (e.g. cases versus controls; exposed (e.g. to dietary, environmental risk factors etc.) versus unexposed; randomised to an intervention or not) [9], [26], [27], [28]. This requires samples to have been collected and processed identically. It also often employs complex multivariate statistics, such as Principal Component Analysis (PCA) or Orthogonal Partial Least Squares – Discriminant Analysis (OPLS-DA). The spectral features responsible for the differences between the samples are then identified so that validation studies and biological interpretation can be performed [13]. This is the approach that, up to recently, had been taken by the majority of researchers using ^1^H NMR metabolomics. An exemplar method, pioneered by Nicholson, is described by Dumas et al., 2014 [26]. Whilst this method can identify patterns of disease, and potentially separate cases from non-diseased controls, the complexity of the clinical interpretation required means it is difficult to relate these findings to traditional studies using routine biochemical measures. Although such techniques, with pattern recognition, can be used to diagnose disease (without necessarily knowing the identity of the metabolite changes), researchers are becoming increasingly aware of the need to identify metabolites contributing to the altered region of the spectra in order to provide transparency and meaningful biological context [29]. Clinicians generally want to see these results in clinically meaningful (SI) Units.
2. **Quantitative**^**1**^**H NMR Metabolomics** (also known as metabolite profiling) involves quantifying a targeted set of metabolites [30]. Generally, identification and quantification are obtained with reference to a library of reference spectra of pure compounds [30]. One of the benefits of using identified metabolites (as opposed to metabolite fingerprinting) is that artefacts and metabolites affected by differences in sample collection or the spectral interpretation are less likely to be misidentified as discriminating between cases and controls [31], [32]. Other qNMR methods include Advance Lipoprotein Profiling (ALP) [33]: the quantification of lipoprotein subclasses (their particle concentration and mean size). Another example is the method developed by Finnish researchers (referred to in this review as the Finnish method) and now widely published on by this group and related collaborators [34], [35]. It combines ALP (described below) and qNMR of a number of metabolites and reports molar concentrations of each.

### 2.4 Advanced lipoprotein profiling (ALP) by ^1^H NMR

Lipoproteins are thousands of times bigger than conventional metabolites and so their quantification does not come under the strict definition of metabolomics. However, due to their importance in disease, ALP is worthy of investigation within qNMR.

The need for more detailed lipid analysis, in particular in lipoprotein subparticles, has been a key goal with regard to CVD risk determination [36], [33]. For instance, it is known that two people with the same Low-density lipoprotein-cholesterol (LDL-c) concentration can have discrepant LDL particle (LDL-p) concentrations due to variability of particle size and cholesterol content [37]. Small and large LDL particles may play distinct roles in driving vascular disease [38]. In Multi-Ethnic Study of Atherosclerosis (MESA) participants with discordant LDL-p compared to LDL-c, LDL-p was more strongly associated with carotid intima-media thickness (cIMT) and CVD events than LDL-c (Table 2). HDL subclasses have also been linked to CVD risk, although the findings are more controversial, as recently reviewed by Superko et al., 2012 [39].

**Table 1:** Overview of a subset of relevant studies where serum/plasma ^1^H NMR was used in the investigation of CVD.

Study and brief design description	Numbers	Main findings	Method and reference
**Interventional and experimental studies**			
RCT in patients with T2DM and CHD given rosiglitazone or placebo for 16 weeks.	51 (25 rosiglitazone and 26 placebo)	↑ glutamine and ↓ lactate on rosiglitazone; see Table 2 for effects on lipoprotein subfractions.	Finnish; Badeau et al., 2014 [78]
Patients with angioplasty balloon-induced transient coronary occlusion	30 (20 patients and 10 controls); validation study of 30 patients with chest pain but normal ECG and TnI	At 10 min: ↑ glucose, lactate, glutamine, glycine, glycerol, phenylalanine, tyrosine and phosphoethanolamine; ↓ choline-containing compounds and triglycerides; changes in total, esterified and non-esterified fatty acids; at 10 min ↓ leucine, isoleucine and alanine, but returned to baseline at 120 min; ↑ creatine after 120 min	Metabolite fingerprinting; Bodi et al., 2012 [27]
Exercise induced ischaemia in patients with suspected stable CHD.	31 (22 subjects with exercise induced ischaemia and 9 controls)	↑ glucose, lactate, valine, leucine, isoleucine and methyl and methylene signals from lipids in exercise induced ischaemia. The model correctly predicted 21/22 with ischaemia but wrongly classified 4/9 patients without.	Metabolite fingerprinting; Barba et al., 2008 [79]
**Observational studies**			
Healthy individuals followed up for a median of 5.4 years	9843 adults; validated in 7503 adults	4 biomarkers (AGP, albumin, VLDL particle size and citrate) predicted all-cause mortality (including death form CVD causes) after adjusting for age, sex and conventional risk factors. A biomarker summary score improved AUROC for prediction of mortality in FINRISK from 0.80 to 0.83.	Finnish; Fischer et al., 2014 [80]
Myocardial energy expenditure (MEE) and ^1^H NMR metabolite profiling in HF patients.	61 (46 HF patients and 15 age-matched controls)	↑ 3-hydroxybutyrate, acetone and succinate in patients with increasing MEE (low, intermediate or high)	Metabolite fingerprinting; Du et al., 2014 [28]
Same-sex twin pairs with one active and one sedentary twin; 3 population-based cohorts also included.	16 twins pairs 1037 pairs from 3 population cohorts	↑ PUFA compared to saturated FA in sedentary individuals; ↓ isoleucine, AGP and glucose in active individuals.	Finnish; Kujala et al., 2013 [81]
Observational study with cIMT at baseline and 6 years	1573 adults (193 with impaired foetal growth, 1380 with normal foetal growth)	↑ omega-3 FA associated with reduced cIMT progression in impaired foetal growth individuals only.	Finnish; Skilton et al., 2013 [82]
Observational study with cIMT at baseline and 6 years	1595 young adults	Prediction of elevated cIMT was improved by inclusion of ^1^H NMR determined LDL-C, medium HDL concentration, DHA and tyrosine (in place of routinely measured total cholesterol and HDL-c) (AUROC = 0.764 vs. 0.737)	Finnish; Wurtz et al., 2012 [54]
Patients with ischaemic stroke vs. healthy controls; cross-sectional study	101 (54 with stroke, 47 controls)	↑ lactate, pyruvate, glycolate and formate, ↓ Glutamine and methanol in ischaemic stroke	Metabolite fingerprinting; Jung et al., 2011 [83]
Patients with stable carotid atherosclerosis vs. controls; cross-sectional	19 (9 cases, 10 controls)	↑ acetoacetate, creatinine and 3-hydroxybutyrate, ↓ formate, alanine and proline; changes associated with measures of insulin resistance	Metabolite fingerprinting by ^1^H NMR and GC–MS; Teul et al., 2009 [63]
Hypertensive patients vs. controls; cross-sectional	80 (40 patients with hypertension and 40 normotensive controls)	AGP, choline or choline containing metabolites, urea and an unknown CH_2_–CH group associated with hypertension.	Metabolite fingerprinting; De Meyer et al., 2008 [84]
Observational study of RCT cohort of T2DM patients with microalbuminuria/proteinuria followed up for 4 years	190 (95 cases of MI or sudden death vs. 95 controls)	Together with lipoprotein deconvolution, spectra were found to be poorly predictive for CVD in these patients, but may add value to classic CVD risk calculations	Metabolite fingerprinting; Roussel et al., 2007 [85]

Metabolite fingerprinting refers to ^1^H NMR with multivariate statistical analysis [9], [70]; the Finnish method is that of the Ala-Korpela group which performs both ALP and qNMR on the same sample (see Table 2 for ALP) [35], [42].

Abbreviations: AGP – alpha-1-acid glycoprotein; AUROC – area under receiver operating characteristic curve; cIMT – carotid intima-media thickness; DHA – docosahexaenoic acid; ECG – electrocardiogram; FA – fatty acid; HF – Heart Failure; MEE – myocardial energy expenditure; MI – myocardial infarction; PUFA – polyunsaturated fatty acid; RCT – Randomised controlled trial; T2DM – type 2 diabetes mellitus, TnI – Troponin I.

**Table 2:** Overview of a subset of relevant studies where ALP of serum/plasma using ^1^H NMR was used in the investigation of CVD.

Study and brief design description	Numbers	Main findings	Method and reference
**Interventional and experimental studies**			
Dietary intervention with fatty fish, lean fish or lean meat for 8 weeks in patients with CHD	33 (11:fatty fish; 12: lean fish; 10: lean meat)	↑ ω-3 FA (including DHA), mean HDL-size and HDL content (total lipid, cholesterol and cholesterol ester) in the fatty fish group.	Finnish; Erkkila et al., 2014 [86]
RCT in patients with T2DM and CHD given rosiglitazone or placebo for 16 weeks.	51 (25 on rosiglitazone; 26 on placebo)	Rosiglitazone did not change lipoprotein profile; trends towards ↑ large-HDL-lipid, large HDL-c and very small VLDL-lipid observed. See Table 1 for effect on metabolites.	Finnish; Badeau et al., 2014 [78]
Dietary intervention for 12 weeks in patients with metabolic syndrome	105 (37 on ‘healthy’ diet, 34 whole-grain diet, 34 control diet)	↑ ω3 FA, DHA and PUFA on healthy diet; Greatest increase in fish intake was associated with ↑ large HDL-p, ↑mean HDL-size and HDL-lipid content	Finnish; Lankinen et al., 2014 [51]
RCT of intense lifestyle change or metformin to reduce new-onset DM in patients with IGT	1645 high DM risk individuals	Metformin: ↓ small dense LDL, ↑ small and large HDL; intensive lifestyle: ↓ large buoyant VLDL, small dense LDL and small HDL and ↑ large HDL.	LipoScience; Goldberg et al., 2013 [43]
RCT of simvastatin versus placebo in patients at high risk of CVD followed up for 5.3 years	20,021 adults	All 4 measures of LDL (LDL-c, non-HLD-c, LDL-P and ApoB) were equally strong predictors of CVD events in both the placebo and statin groups. Additional subparticle quantification did not add value; HDL-p/LDL-p and HDL-c/LDL-c were equally associated with risk (after adjusting for LDL-p).	LipoScience; Parish et al., 2012 [87]
Nested case control analysis of RCT investigating oestrogen and progesterone in postmenopausal women	708 (354 women with early CHD event, 354 controls)	HRT: ↑ HDL-c (*p* ≤ 0.00^1^) and HDL-p (*p* ≤ 0.001), ↓ LDL-c (*p* ≤ 0.001), but did not lower LDL-p.	LipoScience; Hsia et al., 2008 [88]
Nested case control analysis of RCT investigating gemfibrozil for secondary CVD prevention over 5.1 years	1061 (364 men with CVD event, 697 controls)	Gemfibrozil: ↑ HDL-c by 6%, no significant change in LDL-c, ↑ LDL size by 2%, ↓ LDL-p by 5% (especially small LDL-p (↓ by 20%), ↑ HDL-p by 10% (especially small HDL-p (↑ by 20%), no significant change in mean HDL size. A 1 SD ↑ in LDL-p was an independent risk factor for new CHD event (OR = 1.28 (95%CI 1.12–1.47). A 1 SD ↑ in HDL-p was protective against new CHD events (OR = 0.71 (95%CI 0.61–0.81). The ratio of LDL-p: HDL-p was also significantly associated with CHD events (highest quartile vs lowest quartile RR = 2.4 (95%CI 1.8–3.3).	LipoScience; Otvos et al., 2006 [89]
**Observational studies**			
Initially healthy women with 17 years follow up	27,533 women	24.3% of patients were discordant of LDL-c compared to LDP-p (defined by median cut-offs). Risk was underestimated by LDL-c in LDL-c < LDL-p discordant patients (HR 2.32 (95%CI 1.88–2.85). Risk was overestimated by LDL-c in LDL-c > LDL-p discordant patients (HR 0.42 (95%CI 0.33–0.53)).	LipoScience; Mora et al., 2014 [90]
Individuals with no history of CVD followed up for 10 years	1981 (145 cases, 1836 controls)	A computational model was used to calculate “lipoprotein metabolism indicators” (measures of lipoprotein production, lipolysis and uptake). “VLDL extra-hepatic lipolysis indicator” and “VLDL hepatic turnover indicator” improved risk prediction when combined with HDL-c and LDL-c compared to conventional risk factors (AUROC of 0.795 and 0.812 for conventional and improved models respectively).	LipoScience; Van Schalkwijk et al., 2014 [91]
Patients with CAD and coronary artery stenosis with low baseline HDL-c	160 adults	Small LDL-p correlated with CAD progression (% stenosis), independently of traditional lipoprotein measures.	LipoScience; Williams et al., 2014 [50]
Change in ALP association with change in ^1^H NMR derived fatty acid concentrations over 6 years.	665 adults	Baseline ω3 FA (% total FA) associated with ↓ mean VLDL-size and ↑ mean HDL-size. Baseline ω6 FA associated with ↓ VLDL-size and VLDL-p; ↑ LDL-size and ↑ HDL-size. ↑ in ω3 FA was modestly correlated with ↓ in VLDL-size. ↑ in ω6 FA was correlated with ↓ in VLDL-p and size and ↑ in LDL-size.	Finnish; Mantyselka et al., 2014 [92]
Observational study of high CVD risk patients followed up for 36 months	15,569 high CVD risk patients .	Patients with established CVD or DM who achieved LDL-p <1,000 nmol/L had lower CVD risk (HR 0.75 (95%CI 0.58–0.97) than patients who achieved target LDL-c.	LipoScience; Toth et al., 2014 [93]
Same-sex twin pairs with one active and one sedentary twin; 3 population-based cohorts also included.	16 twins pairs 1037 pairs from 3 population cohorts	Metabolome changes discussed in Table 1. Active individuals: ↓ VLDL and small LDL; ↑ large and very large HDL; ↓ ApoB: ApoA1 ratio; ↓ total TG and VLDL-TG compared to sedentary individuals.	Finnish; Kujala et al., 2013 [81]
RCT of rosuvastatin versus placebo with 1 year follow up.	10,046 asymptomatic individuals	Rosuvastatin: ↑ HDL-p and size (*p* < 0.001). HDL-p was the only measure significantly associated with CVD in the rosuvastatin treated arm (after adjustment) and could potentially be used to monitor residual risk after statin therapy.	LipoScience; Mora et al., 2013 [56]
Individuals with T1DM with ∼6 years follow up.	3544 adults with T1DM	↑ VLDL-c and VLDL-TG and ↓ HDL-c were associated with ↑ mortality.	Finnish; Makinen et al., 2013 [94]
Observational study of weight change over a mean of 6.5 years	683 adults	Individuals with >5% body weight loss: ↓ in apo-B containing subclasses and ↑ large HDL-p. Individuals with >5% body weight gain: ↑ apo-B containing subclasses and ↓ total and medium HDL-p. Strongest correlation between weight change and ALP was with VLDL-p and HDL-size (*r* = 0.28 and −0.32 respectively).	Finnish; Mantyselka et al., 2012 [95]
Observational study with cIMT at baseline and 6 years	1595 young adults	See Table 1 for prediction based on combined lipoprotein and metabolite concentrations.	Finnish; Wurtz et al., 2012 [54]
Observational study of CHD and cIMT over 6 years of follow up.	5598 adults	A 1 SD ↑ in HDL-p was protective against CHD, even after adjusting for LDL-p and HDL-c. (HR 0.75 (95%CI 0.61–0.93)). A similar pattern was seen with cIMT associations.	LipoScience; Mackey et al., 2012 [96]
Observational study of CHD and cIMT over 6 years of follow up.	5598 adults	Patients with discordant LDL-p compared to LDL-c were identified. The number of CVD events was highest in those with raised LDL-p and normal/low LDL-c, intermediate in the concordant group and lowest in those with raised LDL-c but low/normal LDL-p. LDL-p was more closely associated with increased risk than LDL-c (HR = 1.45 (95%CI 1.19–1.78) and 1.07 (95%CI 0.88–1.3) for LDL-p and LDL-c respectively).	LipoScience; Otvos et al., 2011 [37]
Initially healthy women with 11 years follow up.	27,673 women	CVD events associated with ↓ HDL-size and ↑ VLDL. Small LDL-p and large LDL-p were both associated with ↑ incident CVD (adjusted HR (quintile 5 vs 1) of 1.44 and 1.63 respectively). Baseline ALP results could predict CVD, comparably but not better than standard cholesterol measures (particularly total-c: HDL-c ratio) or ApoB: ApoA1 ratio.	LipoScience; Mora et al., 2009 [57]
Initially healthy individuals with 6 year follow up.	2,223 (822 CAD cases, 1401 controls)	CAD cases: ↓ HDL-p (adjusted OR 0.5 (95%CI 0.37–0.66), for highest vs lowest quartile).	LipoScience; El Harchaoui et al., 2009 [55]
Observational study of T2DM patients with microalbuminuria/proteinuria followed up for 4 years.	190 (95 MI cases, 95 controls)	See Table 1 for results including lipoproteins.	Metabolite fingerprinting; Roussel et al., 2007 [85]
Prediction of CHD death in men with Metabolic Syndrome over 18 years of follow up.	428 (214 CHD deaths, 214 matched controls)	↓ risk of CVD death in those with ↑ medium HDL-p (adjusted OR = 0.70 (95%CI 0.55–0.90). LDL-p (even small LDL-p) was not a long-term risk factor for CHD mortality.	LipoScience; Kuller et al., 2007 [97]
*Non-diabetic* (at baseline) individuals, with median 5.2 year follow up	830 (130 DM, 700 controls)	Pre-diabetic individuals: ↑ VLDL-size and ↑ small HDL-p (adjusted OR for 1 SD ↑ = 1.52 (95%CI 1.23–1.87) and 1.35 (95%CI 1.10–1.67 for VLDL-size and small HDL-p respectively).	LipoScience; Festa et al., 2005 [98]

Two main groups perform ALP: the LipoScience group [41], [43], [33] and the Finnish (Ala-Korpela) group, who perform both ALP and qNMR on the same sample (see Table 1 for metabolites) [35], [42]. See individual references for other studies.

Abbreviations: AUROC – area under receiver operating characteristic curve; DHA – docosahexaenoic acid; FA – fatty acid; HF – Heart Failure; HR – hazard ratio; IFG – impaired fasting glycaemia; IGT – impaired glucose tolerance; MI – myocardial infarction; OR – Odds Ratio; PUFA – polyunsaturated fatty acid; RCT – Randomised controlled trial; RR – relative risk; SD – standard deviation; T1DM – type 1 diabetes mellitus; T2DM – type 2 diabetes mellitus; TC – total cholesterol; TG – triglyceride.

Both the Otvos and Finnish groups independently developed absolute lipoprotein quantification in the early 1990s [40], [41]. The Finnish method now reports 14 lipoprotein subclasses [42]. The LipoScience (Otvos) method reports eight lipoprotein subclasses [33], [43]. Both methods use the resonance of terminal methyl groups arising from phospholipids, cholesterol, cholesterol esters and triglycerides. The Finnish method additionally interrogates the resonance resulting from the multiple methylene groups found on these lipids [33]. The broad resonances arising from these methyl and methylene groups depend on the composition and size of the lipoprotein (Fig. 2). Lipids in small high-density lipoprotein (HDL) particles give resonances at a lower chemical shift (ppm). Conversely lipids in very low density lipoprotein (VLDL) give resonances at a higher chemical shift, with LDL and IDL subparticles in between [33], [44]. Average lipoprotein particle size can also be calculated from the ^1^H NMR spectra [33], [44].

The LipoProfile panel has been commercially available from LipoScience Inc (Raleigh, North Carolina) since 1997 [33], [44]. The LipoProfile test includes three measures; LDL-p and ^1^H NMR determined HDL-c and triglyceride concentration in serum or plasma. It was approved by the US Food and Drug Administration (FDA) in 2008 [45] and the test is covered by some medical insurers in the USA [15]. However, the clinical benefits and the extent to which it is used in US clinical practice remains unclear.

Other ^1^H NMR methods for ALP have also been developed, as reviewed by Mallol et al., 2013 [33]. Of particular note is the use of diffusion-edited ^1^H NMR spectra (which use magnetic gradients to attenuate the signals from small molecules and enhance lipoprotein signals). Currently this method is not considered developed enough for use in clinical applications [33].

Traditional methods for lipoprotein analysis, such as density ultracentrifugation and gradient gel electrophoresis are laborious, costly, time consuming and potentially may have less robust reproducibility. They do not provide as many lipoprotein measures as ^1^H NMR. Krauss et al. introduced and refined a method for lipoprotein profiling based on ion mobility spectrometry (IMS) in 2008/2009 [46], [47], [48]. IMS is a method for separation and detection of ions based on their mobility in a flow of gas, which is directly related to each particle's cross-sectional area. Lipoprotein profiling by IMS is available from Quest Diagnostics [49]. This method compares well with ^1^H NMR, Gradient Gel Electrophoresis and Vertical Auto Profile Ultracentrifugation, in terms of identifying associations with coronary artery stenosis [50]. However it involves a 135-min ultracentrifugation step before analysis and particle loss during sample preparation must be accounted for [46], [47].

One of the major assets of ^1^H NMR metabolomics is its ability to quickly quantify the lipoprotein subclass concentration, as well as determine the total lipid, phospholipid, triglyceride, polyunsaturated fatty acids, total cholesterol, cholesterol ester and free cholesterol content [51]. Therefore many see ^1^H NMR as a significant improvement in ALP.

## 3 ^1^H NMR and prediction of cardiovascular disease

### 3.1 A potential use for metabolomics and ALP in CVD?

There are a number of risk calculators available to predict risk for CVD and determine whether a patient requires pharmacotherapy (statin treatment) and/or lifestyle guidance. These are frequently based on “classical” risk factors derived from epidemiologic studies, such as the Framingham Heart Study. More recent versions (QRISK and ASSIGN) use additional risk factors to improve prediction, such as postcode, a marker of social deprivation, and family history [52]. However, performance of these risk calculators can still be improved [12]. New methods for the identification of new biomarkers, or for measuring classical biomarkers, such as ALP, could add predictive value. In order to do this, it will be necessary to identify new biomarkers that are strongly predictive of CVD and that are not correlated (or very weakly correlated) with established risk factors and which therefore can enhance risk prediction beyond established predictors [53]. Biomarkers that correlate strongly with existing risk factors generally do not appear to meaningfully improve risk prediction algorithms for CVD [3], [4]. An example of this phenomenon is the measurement of Apolipoprotein (Apo) AI and ApoB which, when studied in prospective studies (as opposed to case control studies), were found not to add to risk prediction beyond total-, LDL- and HDL-cholesterol measurements [36].

Improving prediction of CVD and hence being able to stratify people into different levels of risk and tailor treatment to those at highest risk does not require the predictors to be causally related to CVD.

### 3.2 ^1^H NMR metabolomics and ALP studies in CVD

A number of studies relating ^1^H NMR metabolomics and ALP to CVD are presented in Table 1, Table 2. These include intervention studies (with diet, exercise and medication), longitudinal cohorts and case control studies. Most of these studies could be described as early phase and ‘hypothesis generating’. As such the clinical or pathophysiological relevance of many of the findings is still unclear. It is notable that most studies are relatively small in size and most have linked their metabolomics outputs to surrogate CVD markers rather than hard CVD end-points, though such studies are beginning to emerge. Furthermore, few studies have attempted to replicate findings in two or more independent cohorts. Given the early stage of this work, we provide below a selected summary of some of the larger and better-conducted studies. Together these suggest ^1^H NMR may hold some promise for clinical practice, though it is important to emphasise that further studies will be needed to advance ^1^H NMR to the clinical setting.

1. **Predicting CVD**. In a prospective study of 1595 individuals (24–39 years olds) with normal baseline cIMT or plaque score (part of the Cardiovascular Risk in Young Finns Study (YFS)), Wurtz et al., 2012 [54] investigated the ability of ^1^H NMR metabolomics and ALP (using the Finnish method) to predict incident plaque or cIMT ≥ 90th percentile over a mean of 6 years. No single ^1^H NMR biomarker increased prediction compared to established risk factors (age, sex, systolic blood pressure, smoking, glucose, total cholesterol and HDL-c). However a combination of 4 biomarkers did improve risk prediction: namely ^1^H-NMR-determined LDL-C and medium HDL-p, docosahexaenoic acid (DHA) and tyrosine. Comparing established risk factors alone to a model with replacement of enzymatically measured total cholesterol and HDL-c with the four new biomarkers, the Area Under the Receiver Operating Characteristic curve [AUROC] increased from 0.737 (with 95% confidence interval (95%CI) of 0.699–0.775) to 0.764 (95%CI 0.726–0.802), *p* = 0.02 [54]. Whilst these findings are of interest, the next steps would be to (i) externally validate this prediction model; (ii) determine the ability of this model to predict hard CVD endpoints; (iii) compare to models including other novel biomarkers that appear to improve prediction, such as the cardiac biomarkers brain natriuretic peptide and high sensitivity troponin T. In this way, researchers would be testing the ^1^H NMR outputs not only against the established predictors but also the best of the emerging (non-NMR metabolite) biomarkers. A key consideration is the extent to which prediction of hard outcomes is improved and balanced by the cost of the new measurements.
2. **Refining lipid measures for CVD prediction**. The European Prospective Investigation of Cancer (EPIC) - Norfolk study studied 822 healthy participants who developed a first coronary artery event during 6 years of follow-up and 1401 matched controls [55]. They used the LipoScience method to demonstrate 3% smaller HDL-size and 1% lower HDL-p in cases. HDL-p remained independently associated with coronary artery disease (CAD) risk after adjusting for triglyceride, ApoB, C-reactive protein (CRP) and other markers of inflammation (adjusted odds ratio (OR) 0.50, 95%CI 0.37–0.66, comparing the highest to the lowest quarters for the HLD-p distribution). However, adjusting and matching for established CVD risk factors was incomplete: the authors did not control for prevalent DM, BP or LDL-c, all of which were different between cases and controls at baseline. The findings therefore need replication in other independent studies with a more complete assessment of established and other emerging (i.e. cardiac biomarkers) predictors.
3. **On**-**statin treatment lipid measures and CVD**. HDL-size and HDL-p, assessed using the LipoScience ALP method, were compared to classical biochemistry measures of HDL-c and ApoA1 in 10,886 participants without CVD in the JUPITER trial (Justification for the Use of statins in Prevention: an Intervention Trial Evaluating Rosuvastatin) [56]. Over 2 years of follow-up CVD events occurred in 234 participants. Those randomised to rosuvastatin, had a 3.8% increase in HDL-p and a 1.2% increase in HDL-size compared to placebo, providing evidence that ^1^H NMR can detect treatment-induced changes in HDL-p and HDL-size. The associations of the four HDL measures (ApoA1, HDL-c, HDL-size and HDL-p), at 1 year, with CVD were analysed separately in the statin and placebo arms. In patients randomised to placebo, HDL-c, ApoA1, and HDL-p had similar inverse associations with CVD risk: adjusted hazard ratio (HR) = 0.79 (95%CI 0.63–0.98); 0.75 (95%CI 0.62–0.92) and 0.81 (95%CI 0.67–0.97) per 1 SD increase respectively. In patients randomised to rosuvastatin, however, on treatment HDL-p was reported to have a stronger inverse association (HR = 0.73, (95%CI 0.57–0.93) with CVD than HDL-c (HR 0.82, 95%CI 0.63–1.08) or ApoA1 (HR 0.86, 95% CI 0.67–1.10), though the 95% confidence intervals suggest statistical consistency of association between the three biomarkers within each group and also between the two randomised groups. HDL-size was not notably associated with CVD in either group. HDL-p remained associated with CVD after adjusting for HDL-c (HR 0.72 (95%CI 0.53–0.97)). This suggests HDL-p may be a better biomarker for residual risk in statin-treated patients than HDL-c or ApoA1. As with other studies in this field this requires further replication.
4. **Effect of lifestyle intervention and Metformin on lipids**. In the Diabetes Prevention Programme (DPP), participants were randomly assigned to one of three interventions: metformin, placebo or lifestyle intervention [43]. The effect on lipoprotein measures in a subset of these patients was performed using the LipoScience platform. A total of 1654 paired samples (baseline and 1 year post-intervention) were available from the three treatment groups. In the minimally adjusted model (age, sex and ethnicity), metformin (compared to placebo) slightly increased small and large HDL-p, large LDL-p and LDL-size (*R*^2^ = 0.5%, 0.9%, 0.3% and 0.5% respectively); slightly decreased small LDL-p (*R*^2^ = 0.6%) and had no statistically significant (at the conventional 5% level) effect on large VLDL-p. In contrast, lifestyle intervention (compared to the placebo arm) raised large HDL-p, large LDL-p and LDL-size (*R*^2^ = 4%, 1%, and 3.4% respectively) and lowered small HDL-p, small LDL-p and large VLDL-p (*R*^2^ = 2.1%, 3.9% and 1.9% respectively). The decrease in BMI due to metformin or lifestyle intervention, which resulted in decreased insulin resistance and increased adiponectin concentration, accounted for a varying degree of change in some of the lipoprotein measures. Supplementary analysis, adjusting for age, sex, race, adiponectin, body mass index (BMI) and insulin resistance (HOMA-IR), were performed to allow better comparison between the groups and to identify the extent to which changes were independent of weight loss. The results suggested that the intervention effects on BMI contributed importantly to the changes in lipoprotein particle sizes with the possible exception of the effect of metformin on small HDL-p. Although interesting, the clinical relevance of these relatively small changes in lipids requires further clarification.

The foregoing narrative overview of relevant ^1^H NMR studies clearly shows a need for expansion and validation of work in other cohorts. These suggest the potential of this approach but highlight the early stage of NMR metabolomics-CVD research meaning that currently available results do not provide sufficient evidence to influence clinical care.

## 4 Other considerations

### 4.1 Accurate quantitation and standardisation

For ^1^H NMR measures to be used for risk prediction, monitoring and setting treatment goals it needs to be shown that the methodology and computational spectral interpretation are precise, accurate, robust and validated. For epidemiology and clinical research, absolute concentration, preferably in SI units, would be helpful. This allows studies from multiple groups to be easily collated for example for meta-analysis. These concentrations must be traceable and if there is systematic error this must be highlighted so that researchers are able to account for differences in their analyses compared to previous studies by other methods.

This is equally important for ALP. The density of lipoproteins is a continuum; lipoprotein remodelling is a dynamic process [33]. Lipoprotein subclass size varies between research groups, as does the number of subclasses reported, as categorisation of the lipoprotein subclasses is method dependent [57]. This makes it difficult to compare the results of different studies. This lack of standardisation of methods has been described as one of the biggest barriers to the translation of ALP to the clinic [33]. In 2011, Rosenson et al. [58] proposed the development of a standardised nomenclature for HDL subfractions (VL, L, M, S and VS). They describe how this can be used for multiple methods despite them being based on differing physiochemical properties of HDL, however consensus appears some way off.

The benefit of ALP to improve clinical care in a cost-effective way needs to be demonstrated before entry into clinical practice; whilst some support has been given for quantifying LDL-p in the US [15], [45], in most European health care systems this is not the case and we would support waiting for clearer evidence that cost benefit is obtained.

### 4.2 Cost-effective and high-throughput

For large studies with thousands of samples, methods that can achieve high throughput of quality information at low cost are important. ^1^H NMR methods, such as the Finnish method, have been described as high-throughput and potentially cost-effective [54].

The FDA approved LipoProfile test (LDL-p, HDL-c and TG) is now available to the clinical laboratory as the Vantera Clinical Analyser (Agilent Technologies Inc) [33], [59]. This 400 MHz ^1^H NMR has been adapted to easily fit in with routine chemistry sample handling, has built-in sample preparation and spectral deconvolution. The approval of the Vantera will help to make LDL-p a more easily available test, however its capital cost will still limit its availability [49].

## 5 Mass spectrometry (MS)

### 5.1 The theoretical basis of MS

**Fig. 3:**
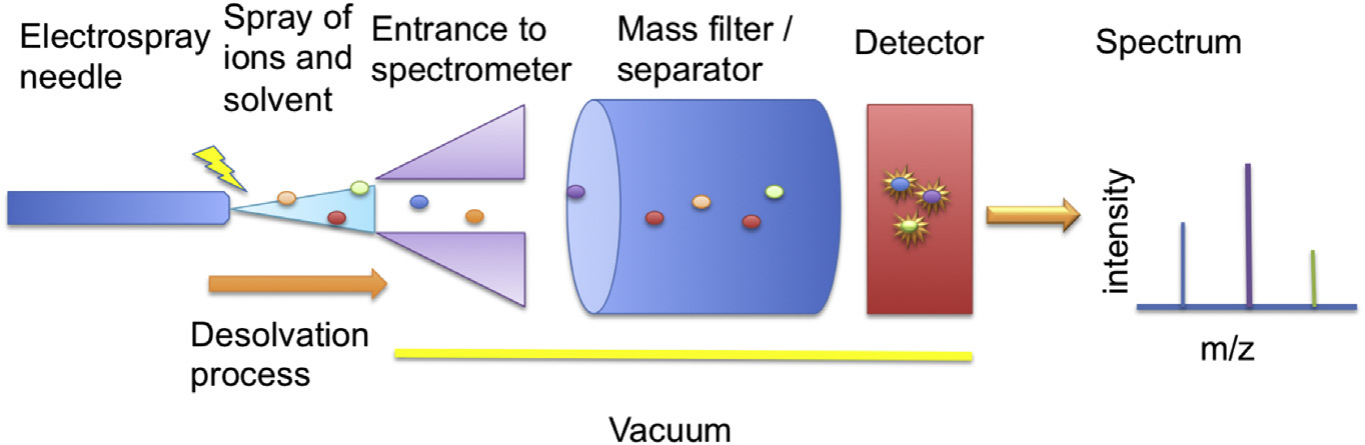
Simplified diagram of a mass spectrometer. Sample, usually in liquid form and eluted from a chromatography instrument, is sprayed using a charged needle and desolvation gas into the high-vacuum interior of the mass spectrometer. Once inside ions may be filtered or separated using a variety of techniques before interacting with a detector. Once separated and detected, a spectrum is produced, graphing mass-to-charge (*m*/*z*) ratio versus the intensity of each ion detected.

MS analysis is based on the detection of ionised molecules and measurement of their mass to charge (*m*/*z*) ratio (Fig. 3). Pre-separation techniques such as capillary electrophoresis (CE) [60], gas chromatography (GC) [61] or liquid chromatography (LC) [62] are routinely used. These separate molecules according to their physio-chemical properties: for example how well they interact with the stationary phase of the column determines how fast they are eluted from the column (their retention time or index). This added information can be used in combination with the *m*/*z* ratio to better identify the metabolites. Another advantage is that instead of infusing a continuous mixture of metabolites into the MS, the eluent from the column is infused over time, meaning that different metabolites are separated because they will be eluted at different times depending on their retention on the column. This reduces the number of different metabolites detected at any time point making the spectra less complex and allowing more sensitive detection of individual peaks (high concentration metabolites are less likely to suppress the signals of low concentration metabolites). This is important because the complexity of biological matrices such as serum is very high. MS is capable of simultaneous detection of very large numbers of metabolites (100s–1000s in some studies) [60].

**Fig. 4:**
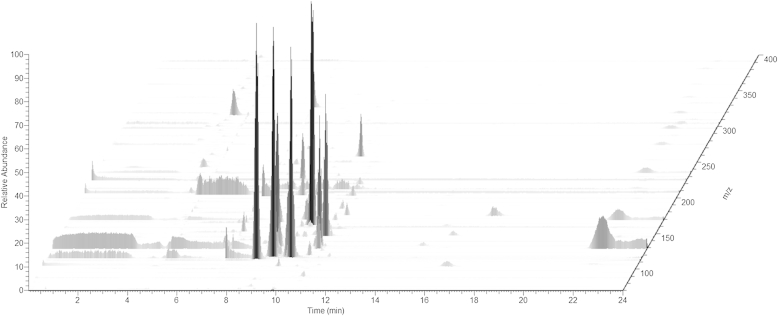
Three-dimensional plot of a typical serum metabolome analysis by untargeted LC-MS. The most intense (in relative abundance on *y*-axis) peaks elute at between 8 and 12 min (*x*-axis) of the separation. The peaks are separated by their *m*/*z* ratio (*z*-axis). Smaller peaks can be observed scattered throughout the analysis. Light grey streaks can be observed crossing the entire duration of the run – these are omnipresent contaminants and can be used for internal calibration. No internal standards are included in this analysis. However, external calibration mix is run several times during a batch.

A typical three-dimensional plot of an untargeted serum MS metabolomics experiment (Fig. 4) depicts the separation of metabolites by their retention time (*x*-axis) and *m*/*z* ratio (*z*-axis). The abundance of each peak is presented on the *y*-axis. It is important to note that the abundances of the peaks cannot be directly used to provide absolute quantification (see below).

## 6 ^1^H NMR versus MS

^1^H NMR and MS are generally described as complementary techniques; they each have benefits and limitations (Table 3). The question of which to use depends on the research (or clinical) objective, the techniques available, the samples themselves and other practical considerations (sample volume, budget). In some cases both MS and ^1^H NMR have been used together to give a comprehensive metabolomic output [63].

**Table 3:** Comparison of ^1^H NMR and MS.

	^1^H NMR	Mass spectrometry
Sample volume	Moderate: 200–400 μL	Small: 10–50 μL
Sample preparation	Simple: add buffer (Sometimes deproteinization by organic solvent or ultrafiltration used)	Simple: varies, e.g. chloroform/methanol/water extraction
Automation	Automated sample preparation and analysis possible	Automated sample preparation and analysis possible
Reproducibility	Very good (sample contained with ^1^H NMR tube so does not contaminate the detector)	Intra- and inter-batch variability has to be corrected for using potentially highly complex QC procedures
Quantification	Absolute quantification routine	Relative quantitation routine Absolute quantification requires IS specific for each metabolite
Throughput	High throughput (few hundred samples per day possible)	Generally lengthy run times required for LC or GC pre-separation
Sample analysis	Non destructive	Destructive
ALP	Useful for lipoprotein profiling	Requires labour-intensive pre-separation
Cost	Generally cheaper due to high throughput but higher capital costs for ^1^H NMR machine	Moderate, generally but commercial costs can be very high
Identification	Identification generally good	Identification often challenging
Data storage	Manageable data sizes	Large data sizes require lots of data storage
Sensitivity (metabolite dependent)	Lower sensitivity (μM)	Higher sensitivity (nM)
Coverage of the metabolome	Smaller numbers of metabolites identifiable (low 100s) due to sensitivity and spectral overlap issues	Huge number of metabolites detectable (100s–1000s)

Main benefits and limitations of ^1^H NMR and MS, in terms of specific attributes, are listed [2], [99]. Note that the summary information provided varies depending on the precise methods of each technique used (see Griffin et al., 2011 [100] for more detailed examples).

The advantages of using ^1^H NMR for metabolomics are that it is high-throughput and can be described as a universal detector [65], [64], as most metabolites have a measurable proton. No chromatographic pre-separation or sample derivatization is required. Together these make ^1^H NMR metabolomics particularly suitable for large-scale epidemiological studies, and routine clinical analysis.

MS is the most sensitive, broad-based method for metabolomics. However, the technique has limitations that have not yet been overcome and that provide challenges for its widespread application to clinical research. These key issues for accurate identification and quantification of large number of metabolites, and how these differ between ^1^H NMR and MS are discussed in more detail below. However, we note that this is a rapidly advancing field with developments to both methods likely to emerge in the short- to medium-term, which will likely reduce these limitations.

1. **Relative or absolute quantitation**: the varying susceptibility of ionisation of each metabolite in MS leads to a specific sensitivity for each molecule, such that the abundance of one metabolite cannot be directly compared to another. Sample to sample comparison of intensity is feasible, and for this reason, relative quantitation (rather than absolute quantitation) is the most common output for MS metabolomic analysis. Absolute quantitation is routinely achieved in MS by the use of a stable isotope labelled IS for each metabolite to be quantified. Kits are available which include multiple IS for quantification, for example the Biocrates kits [66]. However, these are comparatively expensive. ^1^H NMR is an inherently quantitative method, although in practice accurate quantitation requires careful methodological implementation [67]. Importantly, in ^1^H NMR one is always measuring the same variable – the ^1^H signal. Therefore only one IS, for example TMS, is required for all metabolites.
2. **Unambiguous metabolite identification**: One limitation of MS is that, in basing identification of a metabolite on the mass of a compound alone, ambiguous identifications are common. Stringent criteria for metabolite identification [5] state that a metabolite should not be referred to as an “identified metabolite” (as opposed to an “annotated metabolite”) unless two or more orthogonal (based on differing properties e.g. retention time, *m*/*z* ratio and fragmentation pattern) pieces of data match that of an authentic standard. Perhaps the most unique and important feature of ^1^H NMR is that it provides structural information, the chemical shift is dependent on the chemical environment of the ^1^H, which is essentially its molecular structure. However, ambiguous identification can still be a problem, particularly if some resonances are overlapping [68].
3. **Inter**- **and intra**-**batch variability**: MS, unlike ^1^H NMR, requires the sample to physically interact with the instrument. This leads to the build-up of contaminants in the instrument, which can affect the sensitivity. The most effective way of correcting for inter- and intra-batch variation requires the periodic injection of a common pooled sample - a mixture of all the samples in the study [69]. It is then possible to track the variation of signal for any metabolite during the period of analysis and correct for any variation. By contrast, ^1^H NMR has proven reproducible, making it highly suited to large-scale epidemiological studies [70]. Nevertheless, care must be taken in order to achieve this reproducibility (as discussed earlier, sample collection and storage, temperature and other variables must be controlled).
4. **Sensitivity**: In comparison to MS, ^1^H NMR is less sensitive. MS sensitivity depends on the method, instrument, sample type and analyte. Most detection limits are in the nanomolar ranges [71], thus allowing hundreds to thousands of compounds to be detected in a single analysis for high-throughput clinical analysis [69]. The sensitivity of ^1^H NMR is also dependent on the method, instrument, sample and analyte [64], [72], [73]. Cryoprobes and microcoil probes are increasingly used in ^1^H NMR metabolomics to increase the sensitivity [74], [75]. In qNMR sensitivities are normally quoted in the micro-to millimolar range and the number of metabolites detected is normally in the low hundreds [30], [34].
5. **Sample volume**: In comparison to MS, which generally requires only approximately 10–50 μL, ^1^H NMR sample requirements are much higher. A 5 mm ^1^H NMR tube requires approximately 500–600 μL of sample [30] – this volume includes the buffer used so the serum/plasma requirement is typically 200–400 μL. Excessive sample dilution should be avoided due to the sensitivity issues discussed. Microcoil probes are able to analyse approximately 5–30 μL of sample [75], [76].

## 7 Conclusion

^1^H-NMR metabolomics now enables the rapid and accurate measurements of many more metabolites than was previously possible using routine biochemical methods, including detailed analysis of lipoproteins, fatty acids and other metabolic parameters. Whilst MS can measure more metabolites than ^1^H NMR, further work to improve its quality control aspects particularly across batches is needed. The widening availability of ^1^H NMR instruments has led to a rising number of publications in the cardiovascular and metabolic arena, with preliminary evidence that novel lipoprotein measures and metabolites may improve risk prediction of cardio-metabolic disease. While these findings are of interest, they are preliminary and future work is needed to thoroughly assess the clinical and scientific utility of ^1^H NMR spectrometers for predicting disease. In particular, the exploitation of ^1^H NMR metabolomics in larger, prospective observational and intervention studies with meaningful clinical endpoints and large-scale replication is needed. In all such cases, researchers should be cognisant of using robust statistical approaches and should ensure that they compare risk prediction including ^1^H NMR metabolomics to the best available risk prediction algorithms. Parallel work using genetics is needed to tease out potential causal pathways [77]).

## Disclosure

Conflicts of interest: the authors declare they have no conflict of interests.
